# Hydroxysafflor Yellow A (HSYA) Improves Learning and Memory in Cerebral Ischemia Reperfusion-Injured Rats via Recovering Synaptic Plasticity in the Hippocampus

**DOI:** 10.3389/fncel.2018.00371

**Published:** 2018-10-18

**Authors:** Lu Yu, Yanhong Duan, Zheng Zhao, Wendi He, Ming Xia, Qiujuan Zhang, Xiaohua Cao

**Affiliations:** ^1^Comprehensive Department of Traditional Chinese Medicine, Putuo Hospital Affiliated to Shanghai University of Traditional Chinese Medicine, Shanghai, China; ^2^Shanghai Key Laboratory of Brain Functional Genomics, Ministry of Education, School of Life Sciences, East China Normal University, Shanghai, China; ^3^Department of Neurology, Yueyang Hospital of Integrated Chinese and Western Medicine Affiliated to Shanghai University of Traditional Chinese Medicine, Shanghai, China

**Keywords:** Hydroxysafflor yellow A, cerebral ischemia, memory, field potential recording, LTP

## Abstract

Hydroxysafflor yellow A (HSYA) is the major active chemical component of the safflower plant flower, which is widely used in Chinese medicine for cerebrovascular and cardiovascular disease treatment. Recent studies have demonstrated that HSYA exerts neuroprotective effect on cerebral ischemia, such as neuronal anti-apoptosis, antioxidant activity and oxygen free radical-scavenging. However, whether and how HSYA has a protective effect on cognitive impairment induced by cerebral ischemia reperfusion remains elusive. In the present study, by using the middle cerebral artery occlusion (MCAO) model, we found that 8 mg/kg and 16 mg/kg HSYA administration by common carotid artery (CCA) injection improved impaired cognitive function in Morris water maze (MWM) and passive avoidance tasks, but not 4 mg/kg HSYA treatment, suggesting that HSYA treatment in a certain concentration can improve cognitive impairment in MCAO rats. Furthermore, we found that 8 mg/kg HSYA treatment rescued the impaired long-term potentiation (LTP) in hippocampus of MCAO rats. Taken together, these results for the first time demonstrate that HSYA has the capacity to protect cognitive function and synaptic plasticity against cerebral ischemia-reperfusion injury, and provide a new insight that HSYA may be a promising alternative for recovery of cognitive dysfunction after brain ischemic injury.

## Introduction

Stroke is the third leading cause of death and adult disability worldwide, particularly of the elderly (Chen et al., 2012), which is broadly classified as ischemic stroke (approximately 80%–90%) and hemorrhagic stroke (approximately 10%–20%; Goldstein et al., 2006). Ischemic stroke occurs when an artery in the brain is blocked resulting from a transient or permanent reduction in cerebral blood flow (Dirnagl et al., 1999). The stroke patients must not only survive the acute stage of infarction, but also cope with the ongoing neurological impairment. More than half of the stroke survivors experience residual physical disability and cognitive decline (Ivan et al., 2004). Although it has been reported that the impaired sensorimotor function after ischemic stroke could be recovered with time (DeVries et al., 2001; Fluri et al., 2017), cognitive and neuronal dysfunction is irreversible (Bayat et al., 2012, 2015).

The flower of the safflower plant, *Carthamus tinctorius* L. has been widely used in traditional Chinese medicine for cerebrovascular and cardiovascular disease treatment. In the Compendium of Materia Medica, it is described as being able “to invigorate the circulation of blood,” which suggests it has potential benefits for the circulation system (Xu et al., 2012). Hydroxysafflor yellow A (HSYA), first isolated by Meselhy et al. in 1993 (Zhu et al., 2011), is the major active component of safflower which was confirmed that the structure is C-Glycosyl quinochalcones (Black, 2011; Li et al., 2016) and has proven to be water-soluble and penetrative to the blood brain barrier (Meselhy et al., 1993).

Previous studies have demonstrated that HSYA markedly extends coagulation time in mice, which raised the possibility that it might exert therapeutic actives on cerebral ischemia induced by thrombosis (Li et al., 2013). Furthermore, it is confirmed that HSYA could significantly decrease neurological deficit scores, reduce the percentage of infarction following ischemia-reperfusion injury in rats (Sun et al., 2013; Nazari et al., 2016). However, most of researches in this field to date have focused on neuronal anti-apoptosis (Shan et al., 2010), antioxidant activity (Wei et al., 2005) and oxygen free radical-scavenging (Tian et al., 2008) of HSYA in the cerebral ischemic injury. Whether HSYA has benefits on cognitive improvement after ischemia-reperfusion injury is unclear.

In recent years, with more and more studies focus on the neural mechanisms of the cerebral injury, it is demonstrated that brain ischemia impairs physiological form of synaptic plasticity (Feng et al., 2013; Christophe et al., 2017). Clinical symptoms of cognitive impairment such as learning disability, memory loss and lack of executive functioning resulting from ischemic stroke are associated with a loss of synapse number and function in the hippocampus (Jiang et al., 2010). Long-term changes in synaptic transmission, such as long-term potentiation (LTP), are thought to be an indicator of synaptic plasticity at the cellular level (Malenka and Nicoll, 1999; Martin et al., 2000), which is necessary for storage of information by modifying synaptic transmission efficiency. It has been confirmed that LTP can be induced after a short period of brain ischemic injury (Hammond et al., 1994). Also, it has been reported that HSYA improves cognitive function in the rat model of vascular dementia via increasing VEGF and NR1 (Zhang et al., 2014), and enhancing the endogenous expression of BDNF and NMDARs in the hippocampus (Xing et al., 2016). Thus, it is manifested that BDNF and NMDAR play an important role in synaptic plasticity and forming process of LTP in vascular dementia. In addition, it would almost be certain that HSYA has neuroprotective effect on ameliorating cognitive dysfunction. Therefore, it is necessary to study whether HSYA recovers cognitive impairment after brain ischemia via improvement of synaptic plasticity. So far, however, there has been little discussion about the impact of HSYA on synaptic plasticity and memory performance.

In the present work, we adopted middle cerebral artery occlusion (MCAO) model, which is considered to be a convenient, reproducible, and reliable rodent model of cerebral ischemia in humans, to investigate the effects of HSYA on cognitive function and hippocampus synaptic plasticity in cerebral ischemia reperfusion-injured rats. We found that HSYA significantly improved the learning and memory of rats subjected to MCAO model in the Morris water maze (MWM) and passive avoidance tasks. Furthermore, HSYA recovered the impaired LTP at hippocampus while leaving basal synaptic transmission unaffected.

## Materials and Methods

### Animals and Drugs

Sprague-Dawley male rats (weighing 250–290 g, aged 9–11 weeks) were obtained from Shanghai Laboratory Animal Center of Chinese Academy of Sciences. The rats were housed in temperature (22–26°C) and humidity (40%–70%) controlled conditions with a 12/12 h light/dark cycle, and the rats had *ad libitum* access to food and water. All animal experiments described in this study were conducted according to Animals Act (2006; China) and approved by the Institutional Animal Care and Use Committee (IACUC approval ID #M07016) of the East China Normal University. All efforts were made to minimize animal suffering and reduced the number of animals used. In addition, HSYA was produced by Shanghai Yuanye Pharmaceutical Co., Ltd. (Shanghai, China), and its purity as analyzed by high performance liquid chromatography peak area normalization was 98.8%. Solution of drugs was always prepared afresh before use.

### Focal Cerebral Ischemia/Reperfusion Procedure and HSYA Administration

Rats were randomly divided into five groups: (1) sham operated rats; (2) MCAO (vehicle-treated) group; (3) 4 mg/kg HSYA group; (4) 8 mg/kg HSYA group; and (5) 16 mg/kg HSYA group. Focal cerebral ischemia/reperfusion injury was induced by MCAO, based on the method developed by Longa et al. (1989). The rats were anesthetized with an intraperitoneal injection of 10% chloral hydrate (400 mg/kg; Sinopharm Group Chemical Reagent Co., Ltd., Shanghai, China). The right common carotid artery (CCA), external carotid artery (ECA) and internal carotid artery (ICA) were isolated via blunt dissection with a midline incision of the neck. The branches of the ECA were cut off, and the whole of the ECA was ligated. A monofilament nylon suture with a polylysine-coated tip (0.26 mm; Beijing Xinong BioTechnologies Co., Ltd., China) was inserted into the right side of ICA in the depth of 19 ± 0.5 mm from the CCA, to occlude the origin of the (MCA). The monofilament nylon suture was fixed and the incision was closed. Occlusion was done for a period of 1.5 h. After 1.5 h occlusion, reperfusion achieved by withdrawing the monofilament nylon suture to restore blood supply to the MCA territory. After waiting for another 1.5 h, the incision of rat was reopened and HSYA (at a dose of 4 mg/kg, 8 mg/kg, and 16 mg/kg dissolved in 0.9% saline) was injected into the unilateral CCA at a constant speed (0.05 mL/min). The sham-operated rats received all surgical procedures but without the monofilament nylon suture inserted. Body temperature of rats were maintained at 37 ± 0.5°C throughout the surgery by means of a heating blanket and a lamp.

### Evaluation Test of MCAO Model

#### Assessment of Neurological Deficits

Assessment of neurological deficits was performed on rats 24 h after reperfusion by using the Garcia test, which is a neurological examination that utilizes an 18-point scale (Garcia et al., 1995). The examiners were blind to the procedure that the rat had undergone. The neurobehavioral study items included spontaneous activity, symmetry in the movement of four limbs, forepaw outstretching, climbing, body proprioception.

#### Measurement of Edema (Brain Water Content)

Cerebral edema was determined 24 h after reperfusion via measuring brain water content by means of the standard wet/dry weight method (Hatashita et al., 1988). Brains were removed quickly under ice and weighted on an electronic balance to obtain wet weight and then were placed in an oven at (100 ± 2)°C for 24 h to get their dry weight. Brain water content percentage was calculated according to the following formula: [(wet weight − dry weight)/wet weight] × 100%.

#### Measurement of Infarct Volume

Triphenyltetrazolium chloride (TTC) staining was used to measure the infarct volumes 24 h after reperfusion. Rats were killed under deep anesthesia using 10% chloral hydrate and brains were rapidly took out and cut into 2-mm thick coronal sections using the brain matrix (Beijing Xinong BioTechnologies Co., Ltd., China). The fresh slices were incubated away from light in 2% 2,3,5-triphenyl-tetrazolium chloride solution at 37°C for 30 min to visualize in infarctions. Normal and damaged tissue were stained red and white, respectively. The brain slices were photographed with a digital camera and the size of the infarct area (unstained) was assessed by ImageJ software. The percentage of the infarct volume was calculated according to the following formula: {[contralateral volume − (ipsilateral volume−infarct volume)]/contralateral volume}·100%.

### Behavioral Study

#### Open Field

After modeling and drug treatment, the rat was first tested by open field to assess its locomotor and exploratory activity. The apparatus (Gray et al., 1975) was a square black box (100 cm long × 100 cm wide × 50 cm high) and placed under dim light. The rat was placed in the box to explore the arena freely for 30 min, and all activities were recorded using a video camera mounted above the open field and recorded in real time. Move time and total distance were analyzed using the motion tracking system.

#### Morris Water Maze Test (MWM)

The ability of spatial learning and memory was evaluated by Morris water maze (MWM) test (Morris, 1984). All of five groups of rats after open field test were trained and tested in MWM for the purpose of the spatial cognition study. The water maze consists of a large circular pool (150 cm in diameter, 60 cm in height, filled to the depth of 45 cm with water at 22°C ± 1°C). The water is made opaque with black nontoxic ink. The pool is divided into four equal hypothetical quadrants and includes four points in each quadrant (North, East, South and West), plus a hidden circular platform (10 cm in diameter) painted black and submerged 1.5 cm beneath the surface of the water in the southwest quadrant. The position of the platform is unaltered throughout the training session. A digital camera was located above the MWM to record the rats’ swimming pathways.

#### Acquisition Trial

The consecutive training days were commenced from the 4th day after the termination of the HSYA treatment described above. All of rats were trained through four trial sessions in the afternoon each day for five sequential days, and the southwest quadrant was maintained as the target quadrant in all acquisition trials. Facing against the maze wall, the rats were released into the pool randomly in one quadrant (North, East, South or West) for each trial without repetition. They should find the hidden platform according to the program’s instructions. For memory acquisition, the location of the platform remained stable and the rats were given a maximum of 60 s to find the hidden platform. Once a rat climbed onto the platform and remained on it for 20 s, the trial was terminated. If the rat failed to reach the platform within 60 s, it would be gently guided to the hidden platform and allowed to stay on it for 20 s. The average swimming speed was recorded and escape latency time, the time required for the rats to climb the platform was recorded as well. After 5-day acquisition trial, the rat was subjected to the retrieval test in the 6th day.

#### Retrieval Trial

Memory retention was evaluated during a probe test. On the 6th day, the platform was removed from the pool. Each rat was placed in water maze and allowed to swim for 60 s to explore it. Every rat was subjected to one such trial, and each trial was started by placing the rat in the quadrant farthest from where the platform had previously been placed. The platform crossings and the time spent in the target quadrant for searching the missing platform were recorded. The experimenter always stood at the same position. Throughout the study, experimenter must be careful not to disturb the relative location of the pool with respect to other objects in the laboratory, which could be served as prominent visual clues.

#### Passive Avoidance Test

Passive avoidance test (PAT) which was conducted in another batch of rats used for evaluating learning and memory (Loureiro et al., 2012) was executed over 2 days and divided into acquisition trial and retention trial. The apparatus for the passive avoidance study consisted of light and dark chambers (20 cm × 20 cm × 20 cm) with stainless steel bars (2 mm in diameter and 1 cm in distance) on the floor, divided by a guillotine door (5 cm × 5 cm) that separated the two chambers. In the acquisition trial, each rat was placed in the light chamber and the door between the two chambers would be opened 30 s later. After the rat entering the dark chamber, the door was automatically closed, and an electrical shock (0.5 mA) was discharged through the floor grid for 2 s. If the rat did not enter the dark chamber within 60 s after the door opened, it had to be put into the dark chamber, and the latency was considered as 60 s. The retention trial was performed 24 h after the acquisition trial, in which the rat was allowed to enter into the dark chamber freely while the entrance time to the dark chamber was recorded as the step-through latency (STL). However, there was no electrical shock in the retention trial when rats entered the dark chamber. The maximum cut off time for the STL was 300 s.

### Electrophysiological Study

#### Preparation

After the behavioral tests, rats were anesthetized using chloral hydrate and decapitated. The protocols were similar to those described previously (Tang et al., 1999; Duan et al., 2018). Rats were anesthetized with sodium pentobarbital (40 mg/kg, intraperitoneal) and killed by decapitation. The brain was removed immediately. Whole brain was cut into coronal slices (370 μm thickness) containing the hippocampus using a vibroslicer (Vibratome 3000; Vibratome, St. Louis, MO, USA) with cold (4°C) and oxygenated (95% O_2_, 5% CO_2_) modified artificial cerebrospinal fluid (section ACSF) containing (in mM): choline chloride, 110; KCl, 2.5; CaCl_2_, 0.5; MgSO_4_, 7; NaHCO_3_, 25; NaH_2_PO_4_, 1.25; and D-(+)-glucose, 25; pH 7.4. The slices were recovered in an incubation chamber with normal ACSF (incubation ACSF) containing (in mM): NaCl, 119; CaCl_2_, 2.5; KCl, 2.5; MgSO_4_, 1.3; NaHCO_3_, 26.2; Na_2_HPO_4_, 1.0; and Dglucose, 11; pH 7.4, 95% O_2_ and 5% CO_2_ for 60 min at 31°C before recording.

#### Field Potential Recording

For field potential recording, slices were transferred to a recording chamber filled with the oxygenated incubation ACSF, and the rate of ACSF superfusion was 0.5 mL/min. An unipolar tungsten stimulating electrode was placed in the stratum radiatum to activate the Schaffer-collateral pathway projecting to CA1 and the field excitatory post-synaptic potentials (fEPSPs) were recorded using a glass microelectrode filled with 0.1 M CH_3_COONa (3–5 MΩ). A typical experiment began with an input-output curve ranging from subthreshold to maximal response. Fiber volley amplitude was measured from peak negative voltage to baseline. Paired-pulse facilitation (PPF) was assessed among inter-stimulus intervals ranging from 0.02 s to 0.4 s. The PPF was defined as the ratio of the amplitude of the second to that of the first fEPSP amplitude elicited by pairs of stimuli (pulse2/pulse1 × 100). Synaptic responses were monitored with stimuli consisting of constant current pulses of 0.05 ms duration at 0.033 Hz.

After obtaining a stable baseline response for at least 15 min, LTP was induced by theta burst stimulation (TBS; 10 bursts of four pulses at 100 Hz separated by 200 ms). fEPSP continued to be recorded for 60 min after TBS stimulation. Data were recorded using a Multiclamp 700B amplifier and digitized with a Digidata 1322A (Axon Instruments, Foster City, CA, USA). The fEPSP amplitude was used to measure the synaptic efficacy and LTP induction was expressed as the percentage of the increase of fEPSP amplitude compared with the average amplitude of the baseline period. If the change of fEPSP amplitude in sham group of rats exceeded 20%, it was defined as a successful induction of LTP.

#### Statistical Analysis

All data were shown as mean ± SEM. Student’s *t*-test was used for comparison of two groups. For comparisons with multiple data sets, one-way analysis of variance was used. For input-output curve and PPF analysis, two-way repeated measures ANOVA followed was used. Differences were considered statistically significant at *p* < 0.05.

## Results

### Evaluation of MCAO Model

**Figure 1:**
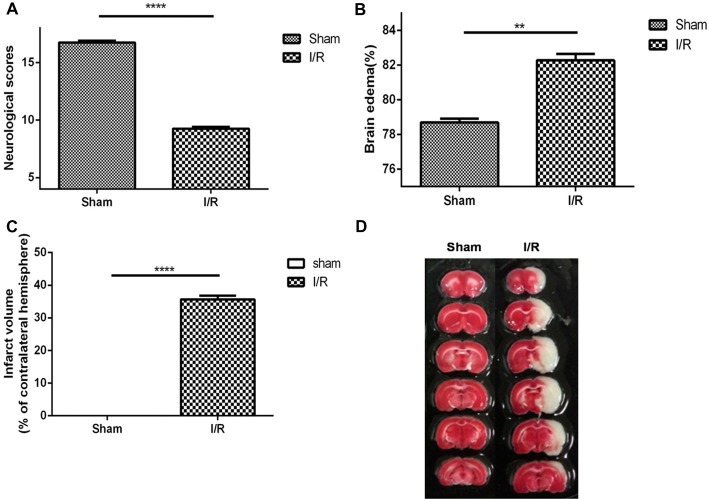
Middle cerebral artery occlusion (MCAO) model was evaluated to be suitable for studying the pharmacology efficacy of Hydroxysafflor yellow A (HSYA). **(A)** Neurological scores, **(B)** brain edema, **(C)** infarct volume, **(D)** pictures of infarct volume. The values are shown as means ± SEM. Significant difference between sham group vs. MCAO group in neurological scores (sham, *n* = 8; MCAO, *n* = 8, ****P < 0.0001), brain edema (sham, *n* = 2; MCAO, *n* = 6, **P < 0.01) and infarct volume (sham, *n* = 2; MCAO, *n* = 8, ****P < 0.0001).

The neurological scores, brain edema and infarct volume were evaluated at 24 h of MCAO. The results showed that neurological scores (Figure 1A, sham, 16.75 ± 0.16, *n* = 8; MCAO, 9.25 ± 0.16, *n* = 8; Student’s *t*-test, *P* < 0.0001) was significantly decreased in MCAO rats. Brain water content (Figure 1B, sham, 16.75 ± 0.16, *n* = 2; MCAO, 82.28 ± 0.37, *n* = 6; Student’s *t*-test, *P* < 0.01) was significantly increased in MCAO rats. Moreover, MCAO rats displayed an obvious manifestation of cerebral infarction (Figures 1C,D sham, 0.00 ± 0.00, *n* = 2; MCAO, 9.25 ± 0.16, *n* = 8; Student’s *t*-test, *P* < 0.0001), whereas rats in the sham group did not show any signs of cerebral injury, indicating the success of the MCAO model.

### Behavioral Experiments

#### Effect of HSYA on Locomotor Activity in the Open Field

**Figure 2:**
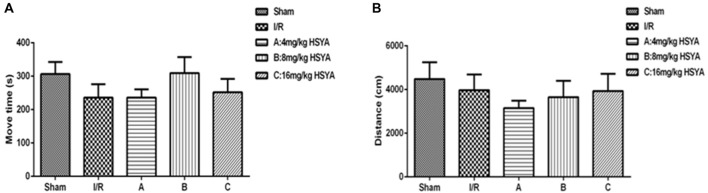
Locomotor activity of HSYA on open field test. **(A)** Move time, **(B)** distance. The values are shown as means ± SEM.

To investigate whether HSYA can improve cognitive impairment after MCAO. We began by exploring modifications of the spontaneous locomotor activity of the different groups of rats. As shown in Figure 2, MCAO did not significantly affect the spontaneous locomotion of rats, as gauged by their move time (Figure 2A, move time: sham, 306.4 ± 95.94 s, *n* = 7; MCAO, 235.7 ± 138.8 s, *n* = 12) and distance (Figure 2B, distance: sham, 4,483 ± 2,177 cm, *n* = 8; MCAO, 3,974 ± 2,582 cm, *n* = 13) in the open field test. Moreover, after HSYA treatment, it also showed that HSYA no matter in which dosage (4 mg/kg, 8 mg/kg, 16 mg/kg) has no effect on the spontaneous locomotor activity of control rats (Figures 2A,B, one-way ANOVA, *P* > 0.05).

#### Effect of HSYA on Brain Ischemia-Induced Memory Impairment in the Morris Water Maze Test

**Figure 3:**
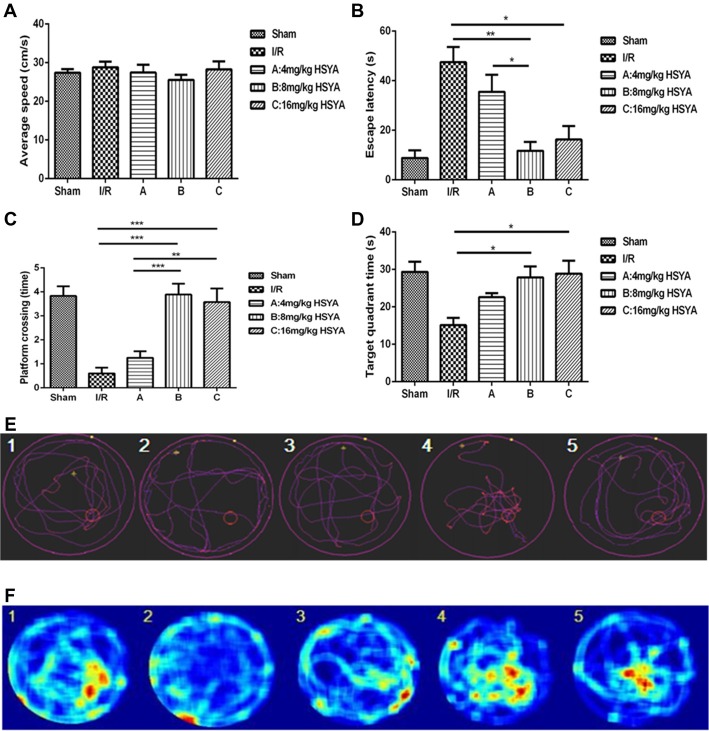
Neuroprotective effects of HSYA on Morris water maze (MWM) test. **(A)** Average swimming speed, **(B)** escape latency, **(C)** platform crossing, **(D)** target quadrant time, **(E)** trajectory of swimming, **(F)** heat-variable images recording rats’ stay in the target quadrant. The values are shown as means ± SEM. Significant difference between MCAO group vs. 8 mg/kg HSYA (*P < 0.05) and 16 mg/kg HSYA groups (**P < 0.01), 4 mg/kg HSYA group vs. 8 mg/kg HSYA group (*P < 0.05) in escape latency; MCAO group vs. 8 mg/kg HSYA (***P < 0.001) and 16 mg/kg HSYA groups (***P < 0.001), 4 mg/kg HSYA vs. 8 mg/kg HSYA (***P < 0.001) and 16 mg/kg HSYA groups (**P < 0.01) in platform crossing; MCAO group vs. 8 mg/kg HSYA (*P < 0.05) and 16 mg/kg HSYA groups (*P < 0.05) in target quadrant time.

To determine the effects of HSYA on the rats’ spatial learning acquisition and memory retention, MWM test was used. All rats learned to locate the hidden platform through 5 days of acquisition training. During this training, we found that the average swimming speed of rats in each and every group was similar, which suggested that every groups of rats have normal sensory-motor function and motivation for survival (Figure 3A, one-way ANOVA, *P* > 0.05). However, the escape latency of 8 mg/kg HSYA group and 16 mg/kg HSYA group significantly decreased compared to that of MCAO group (Figure 3B, MCAO 47.46 ± 6.14 s, *n* = 5; 8 mg/kg HSYA, 11.71 ± 3.61 s, *n* = 9, one-way ANOVA, *P* < 0 0.01; 16 mg/kg HSYA, 16.27 ± 5.42 s, *n* = 7; one-way ANOVA, *P* < 0.05), but not of 4 mg/kg HSYA group (Figure 3B, MCAO, 47.46 ± 6.14 s, *n* = 5; 4 mg/kg HSYA, 35.52 ± 6.91, *n* = 13; one-way ANOVA, *P* > 0.05).

During the retrieval trial, platform crossings and time spent in the target quadrant were calculated for each rat to evaluate memory retention. As shown in Figures 3E,F, the swimmers’ trajectories showed there were fewer platform region crossing times in MCAO group compared to sham group (Figure 3C, crossing times: MCAO, 0.60 ± 0.24, *n* = 5; sham, 3.83 ± 0.40, *n* = 6; one-way ANOVA, *P* < 0.001). After HSYA treatment, 8 mg/kg HSYA group and 16 mg/kg HSYA group produced a significant increase in the retention phase, measured as platform crossings (Figure 3C, MCAO, 0.60 ± 0.55, *n* = 5; 8 mg/kg HSYA, 3.89 ± 0.45, *n* = 9; *P* < 0.001; 16 mg/kg HSYA, 3.57 ± 0.57, *n* = 7; *P* < 0.001), but there was no difference between 4 mg/kg HSYA group and MCAO group (Figure 3C, MCAO, 0.60 ± 0.55, *n* = 5; 4 mg/kg HSYA, 1.25 ± 0.97, *n* = 12; one-way ANOVA, *P* > 0.05). In addition, we found that with the increasing dose of HSYA, rats spent more time in the target quadrant. Target quadrant time in 8 mg/kg HSYA group and 16 mg/kg HSYA group was significant increased compared with that in MCAO group, and even reached values observed in the sham group (Figure 3D, MCAO, 15.13 ± 1.91 s, *n* = 4; 8 mg/kg HSYA, 27.86 ± 2.94 s, *n* = 7, one-way ANOVA, *P* < 0.05; MCAO, 15.13 ± 1.91 s, *n* = 4; 16 mg/kg HSYA, 28.89 ± 3.46 s, *n* = 6; one-way ANOVA, *P* < 0.05). There was still no difference between 4 mg/kg HSYA and MCAO group in time spent in the target quadrant (Figure 3D, MCAO, 15.13 ± 1.91 s, *n* = 4; 4 mg/kg HSYA, 22.58 ± 3.75, *n* = 12; one-way ANOVA, *P* > 0.05). These results suggest that 8 mg/kg HSYA group and 16 mg/kg HSYA can improve the reference memory after brain ischemic injury.

#### Effect of HSYA on Brain Ischemia-Induced Memory Impairment in the Passive Avoidance Test

**Figure 4:**
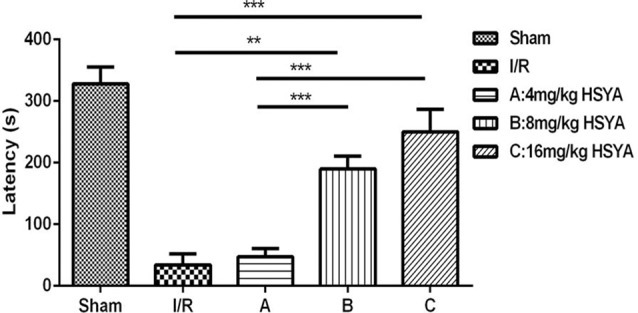
Neuroprotective effects of HSYA on passive avoidance test (PAT). The values are shown as means ± SEM. Significant difference between MCAO group vs. 8 mg/kg HSYA (**P < 0.01) and 16 mg/kg HSYA groups (***P < 0.001), 4 mg/kg HSYA vs. 8 mg/kg HSYA (***P < 0.001) and 16 mg/kg HSYA groups (***P < 0.001) in latency.

To further investigate whether HSYA can improve the cognitive dysfunction after MCAO, passive avoidance task was used. We found that STL significantly declined in MCAO group compared with sham group (Figure 4, MCAO, 34.30 ± 17.90 s, *n* = 3; sham, 327.90 ± 27.79 s, *n* = 6; one-way ANOVA, *P* < 0.001), and 8 mg/kg HSYA group and 16 mg/kg HSYA group produced a significant increase of STL in comparison with MCAO group (Figure 4, MCAO, 34.30 ± 17.90 s, *n* = 3; 8 mg/kg HSYA, 189.80 ± 20.73 s, *n* = 8; one-way ANOVA, *P* < 0.01; 16 mg/kg HSYA, 250.00 ± 36.48 s, *n* = 6; one-way ANOVA, *P* < 0.001), but not 4 mg/kg HSYA group (Figure 4, MCAO, 34.30 ± 17.90 s, *n* = 3; 4 mg/kg HSYA, 47.09 ± 13.74 s, *n* = 14; one-way ANOVA, *P* > 0.05). These data indicate that 8 mg/kg HSYA group and 16 mg/kg HSYA can improve the fear memory after brain ischemic injury.

Taken together, the results of behavioral experiments demonstrated that HSYA treatment in a certain concentration can improve cognitive impairment in MCAO rats. Since the effects of 8 mg/kg HSYA treatment were close to that of 16 mg/kg HSYA treatment, in the following electrophysiological experiments, we selected 8 mg/kg HSYA as an optimal dose to investigate the effects of HSYA on synaptic plasticity.

### Electrophysiological Experiments

#### HSYA Has No Effect on Basal Synaptic Transmission After Brain Ischemic Insult

**Figure 5:**
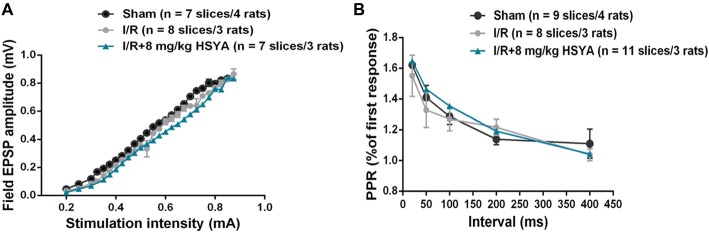
Treatment with HSYA after MCAO did not change basal synaptic transmission. **(A)** HSYA treatment after MCAO have no effect on the input-output curve at Schaffer collateral-CA1 synapses. **(B)** HSYA treatment after MCAO have no effect on the paired-pulse facilitation (PPF) at Schaffer collateral-CA1 synapses.

The cognitive facilitation exhibited by HSYA prompted us to investigate how HSYA facilitates the learning and memory after brain ischemic injury. Given that synaptic plasticity is one of the important cellular foundations of learning and memory, we first examined the basal excitatory synaptic transmission in Schaffer collaterals-CA1 synapse of hippocampus by *in vitro* fEPSP recording. However, no significant difference was observed in input-output curves (Figure 5A: two-way repeated-measures ANOVA, no significant effect of MCAO, *p* > 0.05) and PPF (Figure 5B: two-way repeated-measures ANOVA, no significant effect of MCAO, *p* > 0.05) between sham and MCAO group of rats. In addition, after 8 mg/kg HSYA treatment, there was also no significant difference was observed in input-output curves (Figure 5A: two-way repeated-measures ANOVA, no significant effect of HSYA, *p* > 0.05) and PPF (Figure 5B: two-way repeated-measures ANOVA, no significant effect of HSYA, *p* > 0.05) between HSYA treatment and MCAO group of rats. These results suggest that HSYA have no effect on the basal synaptic transmission after brain ischemic injury.

#### HSYA Rescues Long Term Plasticity Impairment Induced by MCAO Model

**Figure 6:**
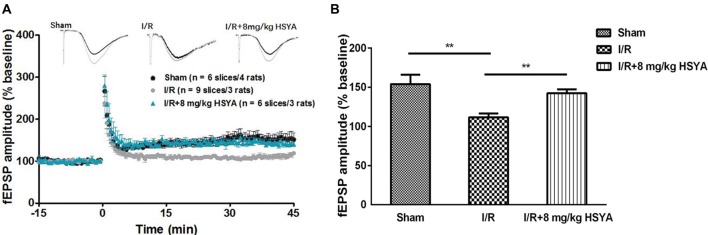
Neuroprotective effects of HSYA on recovery of long-term potentiation (LTP) in MCAO rats. **(A)** Values are expressed as percentage of change relative to baseline. **(B)** The average percent change of field excitatory post-synaptic potential (fEPSP) amplitude 45 min after theta burst stimulation (TBS). The values are shown as means ± SEM. **Represent significant differences before and after tetanic stimulation for sham, MCAO and 8 mg/kg HSYA groups, respectively (**P < 0.01).

We then investigated whether synaptic plasticity in the hippocampus was improved by HSYA treatment after brain ischemic injury. As shown in Figure 6, we compared Schaffer collateral-CA1 LTP induced by TBS among the sham, MCAO and HSYA treatment groups. Forty-five minutes after the TBS, a significant reduction of LTP was found in the MCAO group compared to sham group (Figures 6A,B, sham, 153.89 ± 12.02%, *n* = 6; MCAO, 111.7 ± 4.86%, *n* = 9; Student’s *t*-test, *P* < 0.01). After 8 mg/kg HSYA treatment, the LTP was significantly improved in the HSYA treatment group compared to that in the MCAO group (Figures 6A,B, 8 mg/kg HSYA, 142.28 ± 5.00%, *n* = 6; MCAO, 111.7 ± 4.86%, *n* = 9; Student’s *t*-test, *P* < 0.01); and, there was no statistically difference of LTP between sham group and HSYA treatment group (Figures 6A,B, sham, 153.89 ± 12.02%, *n* = 6; 8 mg/kg HSYA, 142.28 ± 5.00%, *n* = 6; Student’s *t*-test, *P* > 0.05). These data suggest 8 mg/kg HSYA can significantly improve the synaptic plasticity after brain ischemic injury.

## Discussion

Post-stroke cognitive dysfunction is one of the major consequences after stroke and chronic cerebral hypoperfusion can induce vascular cognitive impairment (Back et al., 2017). Currently, thrombolytic drug therapy is used for treating acute ischemic stroke, but the curative effect is limited (Heuschmann et al., 2004). HSYA is a major active component of safflower, which is described as being able to improve the circulation of blood for cerebrovascular and cardiovascular disease. MCAO in rodents is a well-known model of cerebral ischemia in humans to evaluate the progression of impaired spatial learning and memory performance after ischemic stroke (Li et al., 2013). In the present study, we firstly verified the applicability and stability of MCAO model for pharmacological study by assessment of neurological deficits, measurement of brain water content and infarct volume. Then, using this MCAO model, we found that HSYA has the capacity to protect cognitive function and synaptic plasticity against cerebral ischemia-reperfusion injury.

To study whether MCAO model induces cognitive impairment as reported and HSYA has a protective effect on cognitive impairment induced by cerebral ischemia reperfusion, we first carried out a serious behavioral experiment. Using the open field test, we found brain ischemia induced by MCAO did not influence spontaneous locomotor activities on the rats which was consistent with previous study (Chang et al., 2016; Zhang et al., 2017). However, Katsuta et al. (2003) showed that global ischemia induced in gerbils produced a significant increase in locomotor activity and administration of neuroprotective agents ameliorated locomotor hyperactivity. Carmo et al. (2014) showed that mice subject to pMCAO displayed a lower rearing activity. The inconsistency may be due to the difference in model animals, drug intervention and condition of experiment. In addition, in our present study, doses of HSYA were selected based upon pilot study conducted in our laboratory and from available literature which showed it has neuroprotective effects (Bliss and Collingridge, 1993; Meselhy et al., 1993; Ramagiri and Taliyan, 2016). In the open field test, HSYA treatment after MCAO, no matter in which dose (4 mg/kg, 8 mg/kg and 16 mg/kg), did not affect spontaneous locomotor activities on rats.

MWM is one of the classic behavioral test to study spatial learning and memory (Loureiro et al., 2012). The acquisition of spatial memory was reflected through rats’ performance in training trial while the retrieval of spatial memory was reflected through test trial. Using MWM task, we found MCAO rats have normal swimming speed, but the spatial learning and memory was significantly impaired, which was consistent with previous study (Wei et al., 2018). After HSYA treatment, we found that both 8 mg/kg and 16 mg/kg HSYA treatment could significantly reduce the escape latency during acquisition trial, and increase platform crossings and target quadrant time during retrieval trial on MCAO rats, but not 4 mg/kg HSYA treatment. In addition, there was no significant difference between 8 mg/kg and 16 mg/kg HSYA on protection of spatial learning and memory impairment after MCAO. All of these results suggest that HSYA in a certain concentration can improve the impaired spatial learning and memory after brain ischemia.

Passive avoidance is a behavioral test to study fear learning and memory. Using this PAT, we found that the fear learning and memory was significantly impaired on MCAO rats, which was in agreement with previous study (Yang et al., 2015). After HSYA treatment, we found that both 8 mg/kg and 16 mg/kg HSYA treatment could significantly prolong the latency into the dark box on MCAO rats, but not 4 mg/kg HSYA treatment. In addition, there was no significant difference between 8 mg/kg and 16 mg/kg HSYA on protection of fear learning and memory impairment after MCAO. All of these results suggest that HSYA in a certain concentration can improve the impaired fear learning and memory after brain ischemia.

Through behavioral experiments, we found HSYA in a certain concentration improved cognitive function in MCAO rats. The cognitive improvement exhibited by HSYA treatment prompted us to further investigate the mechanism underlying the improvement by HSYA treatment in MCAO rats. Given that synaptic plasticity is one of the important cellular foundations of learning and memory (Esmaeili Tazangi et al., 2015), we first examined the basal excitatory synaptic transmission in hippocampus CA3-CA1 pathway by *in vitro* fEPSP recording. No distinguishable difference was observed between MCAO and sham groups in input-output curves and PPF, suggesting that basal synaptic transmission was unchanged after brain ischemic insult. After HSYA treatment, whether basal synaptic transmission in MCAO rats was effected by HSYA, we found that 8 mg/kg of HSYA which could recover the cognitive impairment after MCAO has no effect on both input-output curves and PPF in MCAO rats. Impairment of synaptic plasticity has been thought of as the mechanism underlying cognitive impairment after stroke (Feng et al., 2013; Christophe et al., 2017). Moreover, the level of long-term potentiation induction is a well-known useful synaptic marker for studying learning and memory ability and is regarded as a basis of information storage (Morris, 1984; Lynch, 2004; Esmaeili Tazangi et al., 2015). Through inducing LTP in hippocampal CA1 region by TBS, we found that LTP at hippocampus was significantly impaired in MCAO group of rats, which was consistent with previous reports (Li et al., 2013; Nazari et al., 2016). It has been reported that HSYA has no effect on hippocampal LTP in sham group of rats (Zhang et al., 2014; Xing et al., 2016). In our present work, after HSYA treatment, we found that 8 mg/kg HSYA treatment could significantly improve the impaired LTP in MCAO rats, and the LTP amplitude almost reached values observed in the control group. Taken together, it suggests that HSYA treatment can recover the synaptic plasticity impairment after MCAO.

It’s known that PPF is an indirect index for measurement of Glutamate release from pre-synaptic terminals (Dobrunz and Stevens, 1997). In our present study, no change was found in the PPF after HSYA treatment, suggesting that HSYA may have no effect on Glutamate release from pre-synaptic terminals. Together with the no changed input-output curves after HSYA treatment, it suggested that both the pre-synaptic transmitter release and the post-synaptic AMPA receptors function were normal after HSYA treatment on MCAO rats. How does LTP impair in MCAO rats, and how HSYA treatment improves this impaired LTP? Using *in vitro* field potential recording, we found that LTP at hippocampus was impaired in MCAO group of rats, which was consistent with previous reports (Li et al., 2013; Nazari et al., 2016). After 8 mg/kg HSYA treatment, the impaired LTP was improved in MCAO rats, and the LTP amplitude even reached values observed in the control group, suggesting that HSYA treatment can recover the impaired synaptic plasticity after MCAO. It has been reported that HSYA can enhance the endogenous expression of BDNF and GluN2B (Xing et al., 2016), VEGF and NR1 (Zhang et al., 2014). It’s well known that all of these three proteins have important role in synaptic plasticity and learning and memory. Therefore, future work will be done to further investigate these protein expressions that related tightly with synaptic plasticity and learning and memory after HSYA treatment. In addition, most recently, LTD was found to couple with synaptic deficits upon aging and AD (Temido-Ferreira et al., 2018). Whether the LTD is also effected by MCAO, we need to detect it in our future work too.

## Conclusion

In summary, we found that MCAO model of brain ischemia induced cognitive impairment and injection with a certain concentration of HSYA into the CCA promoted learning and memory after MCAO. One of the underlying mechanisms of HSYA’s neuroprotective effect is about its capacity of recovery on synaptic plasticity impairment. Thus, our findings of the present study demonstrated that HSYA treatment may be a promising alternative to protect cognitive and synaptic function against brain ischemic injury.

## Author Contributions

LY carried out experiments and wrote the manuscript. YD carried out experiments and revised the manuscript. WH carried out experiments. ZZ analyzed experimental results. MX assisted with data analysis. QZ designed experiments. XC designed and instructed experiments.

## Conflict of Interest Statement

The authors declare that the research was conducted in the absence of any commercial or financial relationships that could be construed as a potential conflict of interest.
